# Role of polygenic and environmental factors in the co-occurrence of depression and psychosis symptoms: a network analysis

**DOI:** 10.1038/s41398-022-02022-9

**Published:** 2022-06-22

**Authors:** Liliana Garcia-Mondragon, Deniz Konac, Joanne B. Newbury, Katherine S. Young, Alex Ing, Anna E. Fürtjes, Edward D. Barker

**Affiliations:** 1 Social, Genetic and Developmental Psychiatry Centre, Institute of Psychiatry, Psychology and Neuroscience, King’s College London, London, UK; 2 International Max Planck Research School for Translational Psychiatry (IMPRS-TP), Munich, Germany; 3 Max Planck Institute of Psychiatry, Munich, Germany; 4 Department of Psychology, Institute of Psychiatry, Psychology & Neuroscience, King’s College London, London, UK; 5 Department of Psychology, Adana Alparslan Turkes Science and Technology University, Adana, Turkey; 6 Centre for Academic Mental Health & MRC Integrative Epidemiology Unit, Population Health Sciences, Bristol Medical School, University of Bristol, Bristol, UK; 7 NIHR Maudsley Biomedical Research Centre, King’s College London, London, UK; 8 European Molecular Biology Laboratory, Heidelberg, Germany

**Keywords:** Depression, Schizophrenia

## Abstract

Depression and psychosis are often comorbid; they also have overlapping genetic and environmental risk factors, including trauma and area-level exposures. The present study aimed to advance understanding of this comorbidity via a network approach, by (1) identifying bridge nodes that connect clusters of lifetime depression and psychosis symptoms and (2) evaluating the influence of polygenic and environmental risk factors in these symptoms. This study included data from European ancestry participants in UK Biobank, a large population-based sample (*N* = 77,650). In Step 1, a network model identified bridge nodes between lifetime symptoms of depression and psychosis and functional impairment. In Step 2, genetic and environmental risk factors were incorporated to examine the degree to which symptoms associated with polygenic risk scores for depression and schizophrenia, lifetime exposure to trauma and area-level factors (including deprivation, air pollution and greenspace). Feelings of worthlessness, beliefs in unreal conspiracy against oneself, depression impairment and psychosis impairment emerged as bridges between depression and psychosis symptoms. Polygenic risk scores for depression and schizophrenia were predominantly linked with depression and psychosis impairment, respectively, rather than with specific symptoms. Cumulative trauma emerged as a bridge node associating deprivation with feelings of worthlessness and beliefs in unreal conspiracy, indicating that the experience of trauma is prominently linked with the co-occurrence of depression and psychosis symptoms related to negative views of oneself and others. These key symptoms and risk factors provide insights into the lifetime co-occurrence of depression and psychosis.

## Introduction

Mental health disorders constitute one of the most challenging global issues of current times, and their impact on society is only expected to increase in coming years [1]. In particular, depression is one of the most prevalent psychiatric disorders worldwide [2], while psychotic disorders are among the most debilitating [3]. When psychosis and depression co-occur, which is quite common [4], treatment and disease burden are further increased [5, 6].

Consistent with high rates of comorbidity, there is common genetic liability underlying both psychosis and depression. Evidence highlights familial aggregation and shared heritability among conditions that include psychotic depression, affective disorders, schizoaffective disorder and schizophrenia [7]. Genome-wide association studies (GWAS) have also shown a substantial shared genetic risk (with a genetic correlation of 0.43) among major depressive disorder and schizophrenia [8].

This observed co-occurrence and shared genetic influence suggests that categorical boundaries between depression and psychosis in current diagnostic classification systems do not fully reflect clinical reality [9, 10], which is further supported by the fact that depression and psychosis have overlapping environmental risk factors. Notable examples include traumatic experiences—in particular the cumulative experience of trauma, which has been consistently associated with increased risk for depression and psychosis [11, 12]—and area-level factors related to urbanicity. In reference to the latter, multiple studies have found increased prevalence of depression and schizophrenia in individuals living in urban areas compared to rural or less populated areas [13, 14], and there is growing evidence of associations between area-level factors that include socioeconomic deprivation, air pollution and greenspace availability with depression and psychosis [15–20].

To advance understanding of the comorbidity between depression and psychosis, alternative views of psychopathology may be warranted. The network approach to psychopathology posits that comorbidities emerge from the interplay between symptoms [9]. Indeed, individual symptoms have been differentially linked to functional impairment and to predisposing risks [21, 22], suggesting that sum scores may obfuscate the understanding of comorbidity. Network analysis allows identification of central and bridge symptoms; central symptoms highlight the interconnectedness within a disorder or symptom network, whereas bridge symptoms can be particularly relevant for explaining comorbidity. These are defined as symptoms that connect clusters of symptoms corresponding to different mental disorders, and thus may play a key role in spreading activation from one disorder to another [9, 23].

Although risk factors are also expected to play an influential role on symptom interactions, only a handful of studies have incorporated them into network models so far [24–26]. In the case of depression and psychosis symptoms, recent network studies have found depressed mood, suspiciousness, delusions, and hallucinations to act as central nodes across symptom networks that include negative, positive, depression and disorganization symptoms and factors related to functional capacity, cognition, motor symptoms, and personal resources [27–30].

Emerging research has also started to uncover the individual influence of genetic and environmental risk factors on depression and psychosis symptoms. For instance, in patients seeking treatment after exposure to psychological trauma, symptoms related to social isolation and negative perceptions of self-worth were identified as bridges between prolonged grief disorder, post-traumatic stress disorder (PTSD) and major depression symptoms [31]. Another recent study found a central role for persecutory-like ideations, feelings of depression and cognitions related to external attribution within a network of traumatic life events, cognitive biases, depression symptoms and psychotic-like experiences in young adults [32]. Finally, an additional network study found associations between a genetic risk score for schizophrenia, notions of conspiracy and paranoia [26].

To our knowledge, there is a scarcity of studies that simultaneously evaluate genetic and environmental risk factors in symptom networks of depression and psychosis. Treating symptoms together with genetic and environmental factors as a dynamic system would enable the detection of clinically relevant bridge symptoms and risk factors linked to them. Identified symptoms and factors could potentially be used as targets for prevention and clinical interventions in the future, as bridge symptoms are expected to play a key role in symptom networks by spreading symptom activation from one disorder to another and potentially generating self-reinforcing feedback loops [33]. Therefore, targeting these symptoms may prevent or reduce symptom activation overall.

The aims of the present study were to identify bridge symptoms connecting lifetime symptoms of depression and psychosis and associated functional impairment in a community sample via a network approach, and to evaluate the influence of polygenic and environmental risk factors in these bridge symptoms. Network models were computed in participants with European ancestry from the UK Biobank study, a large population-based cohort of participants in the United Kingdom aged 40–70 years at the time of the recruitment. UK Biobank contains the largest nationwide urban morphometric database with detailed measures of area-level exposures, as well as one of the largest genotyped cohorts worldwide.

Examining lifetime symptoms in a middle-aged sample enabled a comprehensive evaluation of symptom, functional impairment and risk factor associations in this study, including depression polygenic risk scores (PRS), schizophrenia PRS, cumulative lifetime exposure to trauma and area-level factors consisting of measures of deprivation, air pollution and greenspace availability.

Based on previous research, we hypothesized that feelings of sadness and feelings of worthlessness would be identified as central symptoms in the network [27, 31] and potentially as bridges between depression and psychosis symptoms and risk factors. We also hypothesized that hallucinations and thoughts of conspiracy against oneself would similarly be identified as central symptoms [29, 30] and potentially as bridges in the symptom network. We expected that both genetic risk [34, 35] and the lifetime experience of traumatic events [36, 37] would be associated with functional impairment, and that among the area-level risk factors evaluated, neighborhood deprivation [15, 18] would have the strongest links with depression and psychosis symptoms.

## Methods

### Study sample

UK Biobank is a population-based, observational cohort study that has collected data from ~500,000 participants aged 40–70 years living in the United Kingdom. The UK Biobank study investigates the lifestyle, environmental and biological determinants of a range of adult diseases, and it acquired baseline data between 2007–2010. The study received ethical approval by The North West Multi-Centre Ethics Committee, and all its participants gave informed consent. Details on the setting and study design for the UK Biobank study can be found elsewhere [38].

Participants were selected for the present study if they had completed an online mental health questionnaire (MHQ), which measures the lifetime experience of symptoms of depression, psychosis and traumatic events, had area-level data linked to their residential address available and had genetic data available. Participants who answered “I don’t know” or “I prefer not to answer” to any of the selected questions from the MHQ were excluded from the study.

The MHQ was answered by 157,348 participants at a follow-up assessment completed during 2016–2017. To improve reliability of area-level data, participants were also required to have lived at their address for at least 5 years at baseline to be included in the present study. The sample was further restricted to participants of white European ancestry, as the GWAS used to compute PRS were primarily performed on this ancestry. The final study sample consisted of 77,650 participants.

Power and sample size calculations to estimate appropriate sample sizes for network analyses are not readily available, but preliminary research has found that sample sizes of 250 to 350 tend to produce moderate sensitivity, high specificity and high edge weight correlations in networks with 20 items or less [39]. The considerably large sample available for this study and the number of items included (20 items at most) in our network analyses indicate that our sample size was well-suited for this study.

### Measures

#### Depression symptoms

Self-reported lifetime depression symptoms and related functional impairment were assessed based on items from the MHQ. Evaluation of depression symptoms was based on the lifetime version of the Composite International Diagnostic Interview Short Form (CIDI-SF) [40, 41]. Core lifetime symptoms of depression (“Ever had prolonged feelings of sadness or depression” [low mood] and “Ever had prolonged loss of interest in normal activities” [anhedonia]) were assessed, and if participants endorsed at least one of these, questions related to non-core depressive symptoms (feelings of tiredness, feelings of worthlessness, thoughts of death, weight change, sleep change and difficulty concentrating during the worst period of depression) were queried. Answers to these seven items indicated either the presence (1) or absence (0) of ever having a symptom.

Functional impairment for depression was assessed on a binary scale based on two items from the MHQ: “Impact on normal roles during worst period of depression” and “Professional informed about depression”, which were presented only to participants reporting at least one core lifetime depression symptom. Further details on this measure are presented in the Supplementary Methods.

#### Psychosis symptoms

Self-reported lifetime psychosis symptoms and related functional impairment were also assessed based on items from the MHQ. Evaluation of psychotic experiences was based on the CIDI Psychosis module in its lifetime version [40, 42]. Items included: “Ever believed in an unreal conspiracy against self”, “Ever believed in unreal communications or signs”, “Ever heard an unreal voice” and “Ever seen an unreal vision”. Answers to these four items were also binary (presence = 1; absence = 0).

Functional impairment for psychosis was assessed on a binary scale based on two items from the MHQ: “Distress caused by unusual or psychotic experiences” and “Ever talked to health professional about unusual or psychotic experiences”, which were presented only to participants reporting at least one lifetime psychosis symptom. Further details on this measure are presented in the Supplementary Methods.

#### Traumatic events

Lifetime experience of traumatic events, including childhood trauma, adult trauma and post-traumatic stress disorder (PTSD)-relevant trauma was examined with items from the MHQ. Childhood trauma was assessed by five questions from the Childhood Trauma Screener (CTS), a shortened version of the Childhood Trauma Questionnaire [43], and adult trauma was assessed with an equivalent screener [40]. Both sets of questions evaluated the experience of physical abuse, emotional abuse, physical neglect, emotional neglect and sexual abuse. The experience of events that commonly trigger PTSD was assessed by five items encompassing experiences of violence, accidents, and assault [40].

Items for child and adult trauma were rated on a five-point scale, from “never true” to “very often true”. Items for PTSD-relevant trauma were originally rated on a three-point scale (0—never; 1—yes, but not in the last 12 months; 2—yes, within the last 12 months). As the interest of the present research was on lifetime experience of trauma, and to ensure that the scale of the different trauma measures would have the same range when calculating cumulative trauma, items for PTSD-relevant trauma were recoded to a two-point scale (0—never; 4—yes). Some items on child and adult trauma were also reverse coded so that higher scores represented more severe experience of trauma for all items (Supplementary Table S1). Cumulative trauma experience was computed by summing scores of all trauma questions.

#### Area-level factors

Area-level measures of neighborhood deprivation (measured by the English Index of Multiple Deprivation), air pollution (nitrogen dioxide [NO_2_]) and greenspace availability (measured as the percentage of the living address classed as “greenspace” within a buffer area of 1000 m) were obtained from UKBUMP, a high-resolution spatial database of objective measures of the physical environment surrounding UK Biobank participants’ living addresses [44]. These measures were available on a continuous scale. Further details are presented in the Supplementary Methods.

Area-level factors were available for two timepoints covering the acquisition period of baseline data in the UK Biobank study: 2007 and 2010. Measures from 2010 were selected for this study, as they were acquired at a closer timepoint to the completion of the MHQ in 2016–2017. Within-area-level factor correlations between 2007 and 2010 were highly stable across these timepoints (Supplementary Table S2), lending support to the use of the measures in the present study.

#### Polygenic risk scores

PRS for major depressive disorder and for schizophrenia were generated from UK Biobank genetic data with the use of PRSice [45]. GWAS summary statistics for depression [46] and schizophrenia [47] were obtained from publicly available datasets and used as the base dataset, with UK Biobank genetic data as the target dataset. PRS were created for a *p*-value threshold of 0.1; clumping was set to identify index SNPs and remove SNPs in linkage disequilibrium by using a threshold of *r*^2^ > 0.1 within a 250 kb window. The resulting PRS were on a continuous scale, and each PRS was adjusted for the first five genetic principal components to correct for population stratification.

### Data preparation

Considering available data types for this study (a mixture of binary, ordinal and continuous measures), a binary Ising network approach was chosen, as implemented in previous network studies with a mixture of data types [25, 48]. In contrast with network methods that use continuous measures, this approach does not assume normally distributed measures, which is unlikely to be the case in community mental health data [49]. Cumulative trauma measures were dichotomized at the 75th centile of each measure’s distribution, in the interest of capturing increased non-linear severity of exposure to traumatic events. Each area-level measure and PRS was dichotomized in the same manner. This allowed us to capture increased exposure to neighborhood deprivation, air pollution, greenspace availability and increased genetic risk, respectively.

### Statistical analysis

Two main network models were estimated to examine the interplay of depression and psychosis symptoms with risk factors in an incremental manner. *Step 1* examined the network structure of symptoms of depression and psychosis and associated functional impairment, with 14 items included in the network. *Step 2* incorporated polygenic factors (i.e., depression and schizophrenia PRS) and environmental risk factors (i.e., cumulative trauma and area-level measures) into the depression-psychosis network, with 20 items included in the network. Bridge nodes were identified in both networks. For bridge estimations, depression symptoms and depression impairment were specified as a single community (i.e., a group of predefined nodes measuring similar concepts [50]); psychosis symptoms and psychosis impairment were specified as another separate community, and polygenic factors and environmental risk factors were specified as distinct communities. All analyses were performed with the R-statistical software [51], version 3.6.3.

#### Node selection

To avoid redundancy in the constructs examined by individual nodes (i.e., items in the networks), a node selection step was carried out. The goldbricker function from the *networktools* R software package [52] was used to compare correlations between each pair of variables; node pairs with less than 25% of significantly different correlations and a zero-order correlation of 0.5 or above (*p* < 0.01) would be flagged as redundant. Since no pair of items was identified as such, all measures of interest were represented as individual nodes in the network analyses.

#### Network estimation

Ising network models were used to estimate each of the two networks with the use of the R software packages *bootnet* [53] and *IsingFit* [49]. Ising models generate weighted undirected networks from binary measures. The *IsingFit* package uses an elasso regularization technique that includes model selection based on the extended Bayesian Information Criterion, aiming to identify an optimal network structure that reaches a balance between parsimony and goodness of fit. The product of *IsingFit* is a network whose edges can be interpreted similarly to partial correlations, where an edge/line linking two nodes reflects a statistically significant association after controlling for the remaining nodes in the network. Networks were visualized with the *qgraph* package [54], which uses the Fruchterman Reingold algorithm to place strongly associated nodes closer together and in the center of the network, with weakly connected nodes on the periphery [55].

#### Centrality estimates

Centrality indices of strength, closeness, betweenness and expected influence were computed to evaluate each network and identify nodes with high centrality, which are more theoretically likely to influence network dynamics. Strength measures how strongly a node is directly connected to other nodes, while closeness evaluates how strongly a node is indirectly connected to other nodes, and betweenness quantifies how important a node is in the average path between other pairs of nodes [53, 56]. Similarly to strength, expected influence measures the strength of connections between a node and other nodes, while taking into account the direction of associations between nodes (i.e., positive/negative associations) [57].

In turn, bridge expected influence (Step 2) metrics were calculated to identify bridge nodes, as this metric considers both direct and indirect effects of a node on other communities; nodes with higher levels of this metric most strongly connect multiple communities of nodes. The package *networktools* was used to calculate bridge expected influence (Step 2), and the top 25% scoring nodes on this metric were determined as bridges.

#### Network stability and node centrality

The stability of centrality estimates was evaluated for each network by obtaining case-dropping bootstrapped centrality indices with the *bootnet* package, based on 1000 bootstrapped samples. The *bootnet* package was also used to test the accuracy of network edges with the generation of bootstrapped 95% confidence intervals, and with the use of 1000 bootstrapped samples as well. Only network edges that were present in at least 70% of the bootstraps were reported in this study.

#### Sensitivity analyses

As the lifetime occurrence of some psychosis symptoms had rates that were very low (Table 1), with 96% of the sample reporting no lifetime symptoms of psychosis, networks were recalculated using a down-sampled cohort (*n* = 6222) to assess the possible impact of an imbalanced rate of psychopathology in the network results. This cohort was generated by selecting all participants that had experienced at least one psychosis symptom in their lifetime (*n* = 3111; equivalent to 4% of the total sample), and a matched number of random participants who had never experienced any psychosis symptom in their lifetime. Networks from Step 1 and Step 2 were thus recalculated with data from this down-sampled cohort.

Finally, to evaluate the possible impact of dichotomization of continuous measures in the Step-2 network, this network was recalculated by including the polygenic and environmental risk factors in their continuous scale and the binary symptom measures in a mixed graphical model (MGM). The *mgm* package [58] was employed for the estimation of this network, which produces a regularized model based on extended Bayesian Information Criterion model selection.

## Results

The study sample consisted of 77,650 white European participants with complete data (53% females, mean age at recruitment [SD] = 56.48 [7.52]). Four percent of participants experienced at least one symptom of psychosis during their lifetime, and 1.2% of participants reported significant functional impairment associated with experienced psychosis symptoms. In turn, 42% of participants experienced at least one symptom of depression during their lifetime, and 30% of participants reported significant functional impairment associated with depression symptoms. Table 1 presents the labels assigned to each network node and item frequencies.

**Table 1:** Node labels and node descriptions used in estimated networks, along with item frequencies.

Node	Node description	Item frequency (percentage)
Sad	Feelings of sadness or depression	31,489 (40.6%)
Anh	Anhedonia	23,170 (29.8%)
Tir	Feelings of tiredness	26,402 (34.0%)
Wor	Feelings of worthlessness	16,548 (21.3%)
Dea	Thoughts of death	17,374 (22.4%)
Wei	Weight change	19,702 (25.4%)
Sle	Sleep changes	25,819 (33.3%)
Cnc	Difficulty concentrating	25,289 (32.6%)
Dei	Depression impairment	23,009 (29.6%)
Con	Beliefs in unreal conspiracy against self	467 (0.6%)
Com	Beliefs in unreal communications or signs	435 (0.6%)
Voi	Hearing unreal voices	1,043 (1.3%)
Vis	Seeing unreal visions	2,075 (2.7%)
Psi	Psychosis impairment	951 (1.2%)
Trau	Cumulative trauma	23,346 (30.1%)
IMD	Index of multiple deprivation	19,429 (25.0%)
Pol	Air pollution	19,415 (25.0%)
Gre	Greenspace	19,416 (25.0%)
PRd	Depression PRS	19,442 (25.0%)
PRs	Schizophrenia PRS	19,484 (25.1%)

Total*N* = 77,650.

Network centrality indices were shown to be overall highly stable and therefore reliable in both networks, with most correlation stability (CS) coefficients exceeding the recommended threshold of 0.5 for stable estimation [53]. CS-coefficients for both networks can be found in Supplementary Figs. S1 and S2. In turn, the overall narrow confidence intervals from the edge weight accuracy tests indicated that the estimation of edge weights was generally stable across networks (Supplementary Figs. S3 and S4). Supplementary Table S3 shows the edges that were present in at least 70% of the bootstrapped samples, and which are reported in this study.

### Step 1: Depression-psychosis network

Of the 91 possible edges in the network, 62 (68%) were estimated to be above zero, indicating that the network was highly connected. The mean edge weight in the network was 0.64 units.

#### Symptoms and links within communities

**Fig. 1:**
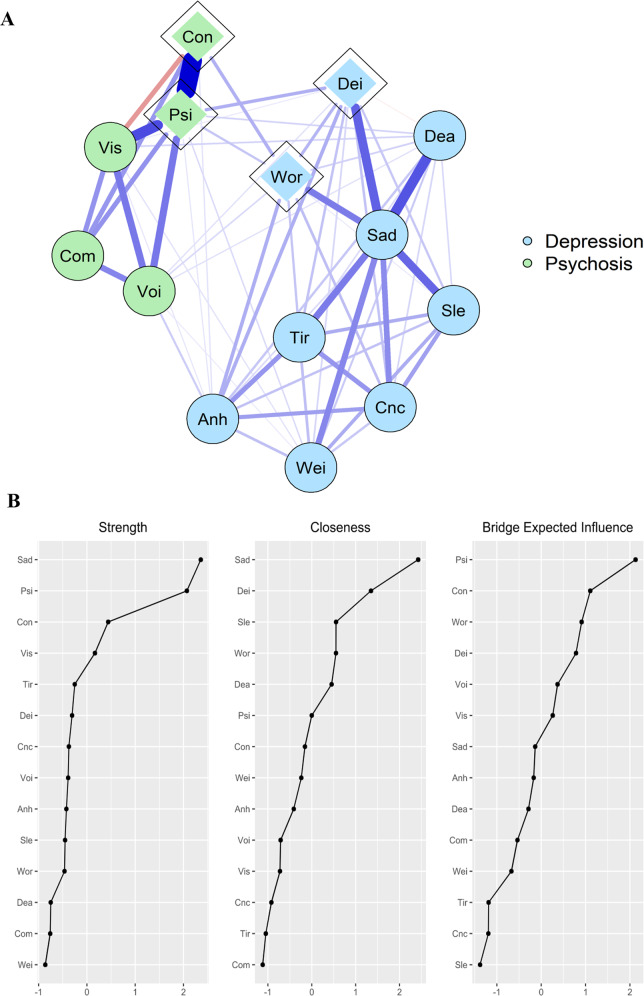
Network of depression and psychosis symptoms in the UK Biobank study. **A** Symptom groups are represented with different colors; bridge nodes are depicted with a diamond shape. Edges (lines) can be interpreted as partial correlations, with edge thickness representing the strength of the correlation. Blue edges represent positive associations; red edges represent negative associations. **B** Centrality indices and bridge expected influence index for Step-1 network. *Anh* = *anhedonia; Cnc* = *difficulty concentrating; Com* = *beliefs in unreal communications; Con* = *beliefs in unreal conspiracy against self; Dea* = *thoughts of death; Dei* = *depression impairment; Psi* = *psychosis impairment; Sad* = *feelings of sadness/depression; Sle* = *sleep changes; Tir* = *feelings of tiredness; Vis* = *seeing unreal visions; Voi* = *hearing unreal voices; Wei* = *weight change; Wor* = *feelings of worthlessness*.

Depression symptoms were strongly connected to each other, and the same was observed for psychosis symptoms. As shown in Fig. 1, *feelings of sadness or depression* had the highest node strength and closeness across the whole network, and it was strongly connected with most depression symptoms. This symptom was also the node with the highest betweenness and expected influence in the network, indicating that this node may activate large portions of the symptom network. *Depression impairment* had its strongest connection with *feelings of sadness or depression*, followed by *anhedonia* and *psychosis impairment*. In turn, *psychosis impairment* was the most central node within the community of psychosis symptoms, showing a high node strength, betweenness and expected influence. This node had strong links with other psychosis symptoms, particularly with *beliefs in unreal conspiracy against self*. Most edges had positive weights, indicating positive associations between symptoms.

#### Links between communities: bridge nodes

As observed in Fig. 1, four nodes were identified as bridge nodes connecting depression and psychosis symptoms: *feelings of worthlessness*, *beliefs in unreal conspiracy against self*, *depression impairment* and *psychosis impairment*. *Feelings of worthlessness* and *beliefs in unreal conspiracy against self* were strongly linked to each other, while the impairment nodes showed strong associations with each other.

### Step 2: Interplay of polygenic and environmental risk factors with depression-psychosis network

**Fig. 2:**
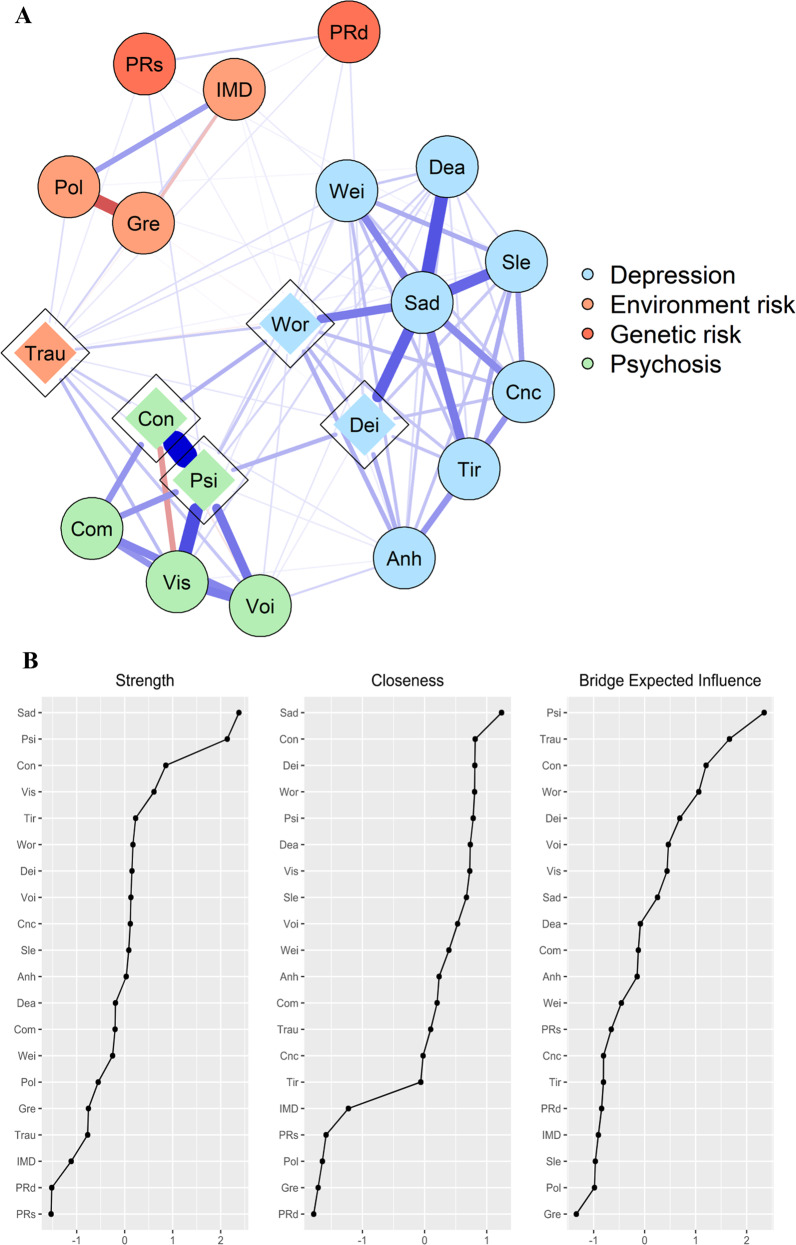
Network of depression and psychosis symptoms with polygenic and environmental risk factors in the UK Biobank study. **A** Symptom groups, polygenic factors, and environmental risk factors are represented with different colors; bridge nodes are depicted with a diamond shape. Edges (lines) can be interpreted as partial correlations, with edge thickness representing the strength of the correlation. Blue edges represent positive associations; red edges represent negative associations. **B** Centrality indices and bridge expected influence index for Step-2 network. *Anh* = *anhedonia; Cnc* = *difficulty concentrating; Com* = *beliefs in unreal communications; Con* = *beliefs in unreal conspiracy against self; Dea* = *thoughts of death; Dei* = *depression impairment; Gre* = *greenspace; IMD* = *Index of Multiple Deprivation; Pol* = *air pollution; PRd* = *depression PRS; PRs* = *schizophrenia PRS; Psi* = *psychosis impairment; Sad* = *feelings of sadness/depression; Sle* = *sleep changes; Tir* = *feelings of tiredness; Trau* = *cumulative trauma; Vis* = *seeing unreal visions; Voi* = *hearing unreal voices; Wei* = *weight change; Wor* = *feelings of worthlessness*.

Of the 190 possible edges in the network, 116 (61%) were calculated to be above zero, and the mean edge weight in the network corresponded to 0.29 units, indicating a lower degree of network connectivity and edge strength compared with the previous network, which did not include genetic and environmental risk factors. As observed in Fig. 2, the addition of genetic and environmental risk factors did not alter relationships within and between depression and psychosis symptoms; rather, it uncovered connections between risk factors and symptoms.

#### Polygenic factors

The *depression* and *schizophrenia PRS* were associated with each other and had sparse associations with the rest of the network; these nodes had low centrality indices in terms of strength, closeness, betweenness and expected influence, which indicates that overall they had a small influence on the symptom network. The *depression PRS* was linked with *depression impairment*, while the *schizophrenia PRS* was connected with *psychosis impairment*. Both PRS were weakly associated with *cumulative trauma* and *feelings of worthlessness* (with stronger links for the *depression PRS*). The *depression PRS* was also weakly linked with *deprivation*.

#### Environmental risk factors

*Cumulative trauma* showed a strong positive connection with *deprivation*, was also positively linked with *air pollution* and it showed a weak and negative connection with *greenspace percentage*. Area-level factors were closely linked to each other, with strong and positive associations between *deprivation* and *air pollution*, and with negative links of *greenspace percentage* with *deprivation* and with *air pollution*. In terms of strength, closeness, betweenness and expected influence, environmental risk factors generally fell midway between symptom nodes (which remained strongly connected to each other) and PRS.

*Cumulative trauma* was the environmental risk factor with the highest centrality indices and with the highest number of links across the network; it was particularly linked with psychosis (*seeing unreal visions*, *hearing unreal voices*, *beliefs in unreal conspiracy against self*) and depression symptoms (displaying its strongest association with *feelings of worthlessness*), and it displayed weaker links with *depression impairment* and *psychosis impairment*. Among the area-level factors, *deprivation* showed stronger connections with depression symptoms, followed in strength by *air pollution*. *Greenspace percentage* lacked any notable associations with depression or psychosis symptoms, in line with its low centrality indices.

#### Bridge nodes

C*umulative trauma* was identified as a new bridge node that mainly linked depression symptoms and psychosis symptoms, including the previously identified bridge symptoms of *feelings of worthlessness* and *beliefs in unreal conspiracy*. The four nodes identified as bridge symptoms in Step 1 (*feelings of worthlessness*, *beliefs in unreal conspiracy against self*, *depression impairment* and *psychosis impairment*) remained as bridges in Step 2. As observed in Fig. 2, *cumulative trauma* showed the second highest bridge expected influence, just below *psychosis impairment*.

### Sensitivity analyses

To evaluate the possibility that an imbalanced rate of presence/absence of psychosis symptoms in the sample could impact network results, symptom networks were recalculated with a down-sampled cohort based on the presence/absence of psychosis symptoms (*n* = 6222). This led to the same pattern of results as in the original sample; the identified bridge nodes remained the same, and most of the network edges observed in the original sample remained. The only exception to this were two edges that were not present in the down-sampled cohort, specifically the links of the *schizophrenia PRS* with *cumulative trauma* and with *feelings of worthlessness*. Visualizations of the down-sampled networks and their centrality estimates are presented in Supplementary Figs. S5 and S6.

Finally, the Step-2 MGM network recalculated with polygenic and environmental risk factors in their continuous scale produced the same pattern of results as the original Step-2 network with binary measures. The identified bridge nodes remained the same, along with most of the network edges observed in the original network. Detailed results for this network can be found in Supplementary Tables S3 and S4.

## Discussion

The present study applied network analysis to investigate the lifetime co-occurrence of individual symptoms of depression and psychosis, and their associations with both genetic and environmental risk factors in a large community sample—one of the largest used in network research to date. To our knowledge, only one other network study has included both genetic and environmental risk factors to investigate psychopathology, albeit in a much smaller sample and with a methodology that could not directly uncover links between symptoms and risk factors [24]. Our study extends current knowledge of the co-occurrence of depression and psychosis symptoms in three main ways.

First, feelings of worthlessness, beliefs in unreal conspiracy against oneself, depression impairment and psychosis impairment emerged as bridges between clusters of depression and psychosis symptoms. This aligns with previous evidence that negative beliefs about the self and others contribute to depression and psychosis symptoms [12, 59, 60], and more generally, with cognitive theories on the role of negative thoughts about the self and others in psychosis [61]. Since the evaluated symptoms in this study are lifetime symptoms, these results mean that individuals who experienced negative beliefs about themselves over their lifetime were more likely to have also had beliefs in an unreal conspiracy against themselves over their lifetime. This study therefore contributes to network studies examining lifetime symptoms [26].

The fact that these symptoms linked depression and psychosis symptom clusters highlights their relevance in lifetime symptom co-occurrence, and places them as possible targets for interventions to reduce or prevent depression and psychosis symptomatology. The relevance of beliefs in unreal conspiracy against oneself in the examined symptom network is further supported by the fact that psychosis impairment had its strongest association with this symptom.

Second, depression and schizophrenia PRS were predominantly associated with functional impairment for depression and psychosis symptoms, respectively, rather than specific symptoms. Of interest, depression and schizophrenia PRS in clinical samples have been correlated with clinical severity and chronicity for depression and schizophrenia, respectively [34, 35, 46]. This is among the first studies to show similar results on a community sample, and it extends findings from previous population-based studies that found no associations between a schizophrenia PRS and symptoms of psychosis [62, 63]. These results suggest that genetic predisposition to depression and to schizophrenia predominantly influences the liability to develop symptoms in a broad, non-specific manner that may affect the frequency, number or intensity of symptoms rather than by influencing the liability to develop specific symptoms.

Third, of the evaluated environmental risk factors, cumulative trauma had the strongest and most extensive connections with depression and psychosis symptoms. Cumulative exposure to traumatic events has been indicated as a key risk factor in the experience of symptoms of depression and psychosis [64], and the number of experienced traumatic events has been suggested to be more important than the specific type of experienced trauma for predicting psychopathology [12, 65]. Interestingly, this risk factor emerged as a bridge node that linked neighborhood deprivation to the bridge symptoms of feelings of worthlessness and beliefs in unreal conspiracy, meaning that individuals living in deprived areas are more likely to have had traumatic experiences over their lifetime, and to have experienced these specific symptoms. In support to this finding, a previous network study found that symptoms of paranoia (i.e., suspicions about others’ intentions) were linked with mental health symptoms in participants living in highly deprived areas [25]. Previous studies have also shown that negative beliefs about the self may partially account for associations between traumatic experiences and paranoia [12, 66].

The experience of trauma, especially when associated with intentions to harm (e.g., physical and emotional abuse), has indeed shown to lead to alterations in cognition and mood such as distorted and long-lasting negative beliefs about oneself and the world, which may make individuals more susceptible to being suspicions about the intention of others [67, 68]. In turn, neighborhood social disorganization and deprivation have been linked with increases in the experience of traumatic events [69, 70], as these contexts are associated with higher social stress, crime and risk of violence. Thus, cumulative trauma may be an important mediator for the relationships between neighborhood deprivation, depression and psychosis symptoms, though prospective studies will be required to disentangle the directionality in these associations.

The results from this study showed distinct associations of genetic and environmental risk factors with depression and psychosis symptoms, with polygenic risk mainly presenting associations with symptomatology through functional impairment, and with cumulative trauma showing specific associations with symptoms related to negative beliefs about oneself and others. These risk factors may therefore confer differential risks for the experience of depression and psychosis symptoms, although additional studies will be needed to further investigate this.

The evaluation of lifetime symptoms of depression and psychosis symptoms allowed to investigate the co-occurrence of these symptoms in a population-based sample, as evaluations over a shorter timeframe (e.g., symptoms experienced within the previous month) may not have been informative enough in this context, given the low incidence of psychosis symptoms in the general population. The application of a regularized network approach in this study allowed to obtain an interpretable network, which is likely to better extrapolate to new samples and which ensures the reduction of false positives [71]. The use of a bootstrapping procedure to evaluate the validity of edge weights across networks and the implemented sensitivity analyses also aided in evaluating the robustness of the results identified in this study. Additionally, the inclusion of a node selection step allowed to identify and remove any pair of nodes that would correlate too highly, which circumvented issues related to potential variance overlap between symptoms and between risk factors included in the networks. The fact that the network approach allows to identify associations between nodes that are present after controlling for the remaining nodes in the network contributed as well to circumventing potential biases in the estimation of symptom-symptom associations and risk factor-symptom associations.

This study is however not absent of limitations. UK Biobank participants who completed the MHQ were on average healthier and of higher socioeconomic status compared with the general population and with the initial cohort of UK Biobank participants [40]. Results from this study may therefore not be fully representative of the general population. Of note, this study evaluated participants with European ancestry only, which may preclude the generalization of results to individuals with a different ancestry. Mood-congruent recall [72] and recall biases in general are another possible source of bias in the identified symptom-environment associations, hence the corroboration of these symptom-risk factor interactions in a shorter timeframe—particularly in clinical samples—will aid in examining the robustness of the results from this study. Overall, research in clinical samples will be needed to evaluate the generalizability of results from this community-sample study, and to determine whether relevant symptoms and risk factors can eventually serve as useful targets for prevention or treatment.

Moreover, studies based on longitudinal data will be necessary to examine whether our results also apply to intra-individual psychological processes over time [73]. Analyses using repeated assessments, such as ecological momentary assessments, will allow to determine the directionality of interactions between symptoms and risk factors identified in this study (e.g., do symptoms related to negative perceptions of oneself activate symptoms related to negative perceptions towards others?) and will aid in assessing whether targeting key symptoms and risk factors have a positive impact on clinical outcomes.

Another potentially relevant limitation of this study relates to our assessment of functional impairment, which was based on two measures: the distress/impact on normal life caused by the experience of depression or psychosis symptoms, and the consultation with a health professional about experienced symptoms. Our choice of evaluating functional impairment by combining these two items could have impacted the observed associations in this study, as results of network analysis depend heavily on the items included. Future studies evaluating functional impairment in alternative ways will be needed to further assess the robustness of results from the present study.

Finally, although the majority of results remained in sensitivity analyses performed on a subsample of participants, a few associations between the schizophrenia PRS and other network nodes were no longer present, indicating that observation of some of these links may be dependent on larger sample sizes (such as our original sample). A broader limitation of the field relates to the low variance that PRS currently explain; as these only partially capture genetic risk, identified associations between PRS and symptoms (and possibly environmental risks) are likely to be underestimates of true genetic-symptom-environment links.

The possible examination of a broader set of psychosis symptoms in future research will also enable a richer characterization of associations between depression and psychosis symptoms. Relatedly, future studies should consider incorporating post-traumatic stress symptoms into the examination of these symptom-risk factor interactions, given the close links between the experience of traumatic life events and of post-traumatic stress symptoms, and the known comorbidity between PTSD, depression [74] and psychosis [75]. The present study was not able to explore the influence of post-traumatic stress symptoms in the examined associations, as information on the lifetime experience of these symptoms was not available in the UK Biobank study. Finally, as specific categories of trauma may be differently associated with psychopathology symptoms [76], relevant insights will be gained if future studies evaluate associations of specific trauma types with depression and psychosis symptoms. For example, there are indications that the exposure to traumatic life events with intention to harm have stronger associations with psychosis symptoms, compared to other traumas (such as accidents) [77].

In conclusion, the present study identified key symptoms and risk factors that give insights into the lifetime co-occurrence of depression and psychosis with the use of a network approach. Present findings point to the experience of symptoms related to negative views of oneself and others and to the cumulative experience of traumatic events as relevant in the co-occurrence of such symptoms. Therefore, pending replication of these results in further clinical studies, efforts to diminish these experiences could potentially aid in reducing or preventing symptoms of depression and psychosis. Policies tackling deprivation at the neighborhood level may also have a positive impact on these symptoms by reducing residents’ cumulative exposure to trauma. Our findings also suggest that in the future, as the predictive capacity of PRS increases, their clinical value might include an ability to prioritize treatment for individuals at highest risk of experiencing functional impairment.

## Supplementary information

Supplementary Information

## Data Availability

Access to UK Biobank data is available to bona fide researchers through a procedure described at https://www.ukbiobank.ac.uk/enable-your-research.

## Code availability

Computer code used to generate results for this study can be made available from the corresponding author upon reasonable request.
