# Supplementary material for: Role of polygenic and environmental factors in the co-occurrence of depression and psychosis symptoms: a network analysis
Source: Transl Psychiatry. 2022 Jun 22;12:259. doi: 10.1038/s41398-022-02022-9 (PMC9217963; doi:10.1038/s41398-022-02022-9)
Supplement: Supplementary file 1 — Supplementary Information [file 41398_2022_2022_MOESM1_ESM.docx]

**Role of Polygenic and Environmental Factors in The Co-Occurrence of Depression and Psychosis Symptoms: A Network Analysis**

**Supplementary Information**

**Supplementary Methods**

**Definition of Depression Impairment**

In this study, functional impairment for depression was assessed based on two items: “Impact on normal roles during worst period of depression” and “Professional informed about depression”. The specific questions related to these items are presented below.

1. "Think about your roles at the time of this episode, including study/employment, childcare and housework, leisure pursuits. How much did these problems interfere with your life or activities?". This question was originally coded as: 0 – not at all; 1 – a little; 2 – somewhat; 3 – a lot.
2. "Did you ever tell a professional about these problems (medical doctor, psychologist, social worker, counsellor, nurse, clergy, or other helping professional)?" This question was originally coded as: 0 – no; 1 – yes.

These questions were asked to participants only if they reported the presence of at least one core symptom of depression (low mood and/or anhedonia) during their lifetime. Depressive symptoms were defined as substantial if participants answered “somewhat” or “a lot” to item 1 or if they answered “yes” to item 2. If this was the case, functional impairment related to depression symptoms was defined as present (1); otherwise it was defined as absent (0).

**Definition of Psychosis Impairment**

In this study, functional impairment for psychosis was assessed based on two items: “Distress caused by unusual or psychotic experiences” and “Ever talked to health professional about unusual or psychotic experiences”. The specific questions related to these items are presented below.

1. "How distressing did you find having any of these experiences (seeing a vision, hearing a voice, or believing that something strange was trying to communicate with you, or there was a plot against you)?". This question was originally coded as: 0 – not distressing at all, it was a positive experience; 1 – not distressing, a neutral experience; 2 – a bit distressing; 3 – quite distressing; 4 – very distressing.
2. Did you ever talk to a doctor, counsellor, psychiatrist or other health professional about any of these experiences (seeing a vision, hearing a voice, or believing that something strange was trying to communicate with you, or there was a plot against you)?". This question was originally coded as: 0 – no; 1 – yes.

These questions were asked to participants only if they reported the presence of at least one psychosis symptom (beliefs in unreal conspiracy against self, beliefs in unreal communications or signs, hearing unreal voices and/or seeing an unreal vision) during their lifetime. Psychosis symptoms were defined as substantial if participants answered “quite distressing” or “very distressing” to item 1 or if they answered “yes” to item 2. If this was the case, functional impairment related to psychosis symptoms was defined as present (1); otherwise it was defined as absent (0).

**Area-level Factors**

The Index of Multiple Deprivation (IMD) for England is a global measure of small-area deprivation, and it was calculated from a weighted combination of seven indicators, which include: income deprivation, employment deprivation, health deprivation and disability, education skills and training deprivation, barriers to housing and services, living environment and crime. These indicators are in turn generated based on national census data. IMD scores are calculated at the lower-layer Super Output Area (LSOA) level, which comprises a small geographic area with an average population of 1,500. LSOA’s were assigned to UK Biobank participants’ living addresses to allow allocation of IMD scores to each participant.

Estimates of air pollution, specifically NO2, were modelled for participants’ addresses with the use of a Land Use Regression (LUR) model, which was developed as part of the European Study of Cohorts for Air Pollution Effects (ESCAPE) (1). The LUR model was based on measured pollutant concentrations at a number of monitoring sites and measures of traffic, land use and topography. Levels of NO2 were measured as annual average values in μg/m^3^.

Finally, measures of greenspace surrounding participants’ living addresses were estimated with the use of the Generalised Land Use database for England (2). This database contains information on land use distribution, with ‘greenspace’ being one of the simplified land categories. A buffer area of 1000 m from participants’ addresses was considered for the estimation of these measures.

**Table S1. Examined traumatic events.**

| Child trauma | Adult trauma | PTSD-relevant trauma |
| --- | --- | --- |
| Someone to take to doctor* when needed as a child | Able to pay rent/mortgage as an adult* | Witnessed sudden violent death |
| Felt loved as a child* | Been in a confiding relationship as an adult* | Been in serious accident believed to be life threatening |
| Felt hated by family member as child | Belittlement by partner or ex-partner as an adult | Been involved in combat or exposed to war zone |
| Sexually molested as a child | Sexual interference by partner or ex-partner without consent as an adult | Victim of sexual assault |
| Physically abused by family as child | Physical violence by partner or ex-partner as adult | Victim of physically violent crime |

**These items were reverse-coded.*

**Table S2. Correlations of area-level factors between the years 2007 and 2010.**

| Measure | *r* coefficient |
| --- | --- |
| Index of Multiple Deprivation | 0.99* |
| Nitrogen dioxide air pollution | 0.79* |
| Greenspace percentage | 0.97* |

**Correlation is significant at a p < 1x10^-3^*

**Table S3. Significant edges in networks: non-zero edges present in at least 70% of a total of 1,000 bootstrapped samples in Step-1 and Step-2 networks and non-zero edges present in Step-2 MGM network.**

| Step-1 Network | | |  | Step-2 Network | | | |
| --- | --- | --- | --- | --- | --- | --- | --- |
| Edge | Edge weight | Presence in bootstrapped samples (%) |  | Edge | Edge weight | Presence in bootstrapped samples (%) | Presence in MGM network (edge weight) |
|  |  |  |  |  |  |  |  |
| Anh--Dea | 0.507 | 100 |  | Anh--Dea | 0.492 | 100 | Y (0.246) |
| Anh--Dei | 0.935 | 100 |  | Anh--Dei | 0.921 | 100 | Y (0.456) |
| Anh--Tir | 1.424 | 100 |  | Anh--IMD | 0.101 | 95.6 | Y (0.044) |
| Anh--Voi | 0.407 | 100 |  | Anh--Tir | 1.422 | 100 | Y (0.704) |
| Anh--Wei | 0.645 | 100 |  | Anh--Trau | 0.148 | 100 | Y (0.078) |
| Anh--Wor | 0.929 | 100 |  | Anh--Voi | 0.355 | 98.5 | Y (0.157) |
| Cnc--Anh | 1.227 | 100 |  | Anh--Wei | 0.611 | 100 | Y (0.302) |
| Cnc--Dea | 0.283 | 100 |  | Anh--Wor | 0.901 | 100 | Y (0.441) |
| Cnc--Dei | 0.553 | 100 |  | Cnc--Anh | 1.221 | 100 | Y (0.603) |
| Cnc--Sad | 1.7 | 100 |  | Cnc--Dea | 0.284 | 100 | Y (0.152) |
| Cnc--Tir | 1.454 | 100 |  | Cnc--Dei | 0.565 | 100 | Y (0.29) |
| Cnc--Wei | 0.498 | 100 |  | Cnc--Sad | 1.667 | 100 | Y (0.828) |
| Cnc--Wor | 0.646 | 100 |  | Cnc--Tir | 1.425 | 100 | Y (0.713) |
| Com--Psi | 1.417 | 100 |  | Cnc--Wei | 0.487 | 100 | Y (0.252) |
| Com--Vis | 1.552 | 100 |  | Cnc--Wor | 0.632 | 100 | Y (0.32) |
| Com--Voi | 1.823 | 100 |  | Com--Psi | 1.35 | 97.1 | Y (0.745) |
| Con--Com | 1.498 | 100 |  | Com--Vis | 1.506 | 100 | Y (0.692) |
| Con--Psi | 6.368 | 100 |  | Com--Voi | 1.809 | 100 | Y (0.895) |
| Con--Vis | -1.558 | 100 |  | Con--Com | 1.458 | 100 | Y (0.708) |
| Con--Voi | -0.094 | 90 |  | Con--Psi | 6.021 | 97.2 | Y (3.019) |
| Dea--Dei | -0.058 | 70 |  | Con--Trau | 0.416 | 89.8 | Y (0.241) |
| Dea--Psi | 0.353 | 100 |  | Con--Vis | -1.426 | 100 | Y (0.656) |
| Dea--Vis | 0.334 | 100 |  | Dea--Psi | 0.287 | 93.2 | Y (0.151) |
| Dea--Voi | 0.2 | 90 |  | Dea--Trau | 0.185 | 100 | Y (0.093) |
| Dea--Wei | 0.467 | 100 |  | Dea--Vis | 0.279 | 100 | Y (0.134) |
| Dei--Psi | 0.92 | 100 |  | Dea--Voi | 0.133 | 78.2 | Y (0.068) |
| Sad--Anh | 0.164 | 100 |  | Dea--Wei | 0.459 | 100 | Y (0.23) |
| Sad--Dea | 3.2 | 100 |  | Dei--PRd | 0.191 | 100 | Y (0.081) |
| Sad--Dei | 2.839 | 100 |  | Dei--Psi | 0.807 | 97.2 | Y (0.405) |
| Sad--Tir | 2.082 | 100 |  | Dei--Trau | 0.144 | 100 | Y (0.07) |
| Sad--Wei | 1.799 | 100 |  | Gre--IMD | -0.633 | 100 | Y (0.064) |
| Sad--Wor | 2.088 | 100 |  | IMD--PRd | 0.041 | 83.5 | Y (0.013) |
| Sle--Anh | 0.591 | 100 |  | Pol--Gre | -3.158 | 100 | Y (-0.722) |
| Sle--Cnc | 1.183 | 100 |  | Pol--IMD | 1.236 | 100 | Y (0.304) |
| Sle--Dea | 0.269 | 100 |  | PRd--PRs | 0.37 | 100 | Y (0.139) |
| Sle--Dei | 0.493 | 100 |  | Psi--PRs | 0.201 | 79.2 | Y (0.017) |
| Sle--Sad | 2.682 | 100 |  | Psi--Trau | 0.187 | 83.5 | Y (0.147) |
| Sle--Tir | 0.968 | 100 |  | Sad--Anh | 0.229 | 100 | Y (0.393) |
| Sle--Wei | 0.97 | 100 |  | Sad--Dea | 3.191 | 100 | Y (1.612) |
| Tir--Dea | 0.239 | 100 |  | Sad--Dei | 2.818 | 100 | Y (1.413) |
| Tir--Dei | 0.578 | 100 |  | Sad--Tir | 2.09 | 100 | Y (1.044) |
| Tir--Wei | 0.62 | 100 |  | Sad--Wei | 1.797 | 100 | Y (0.886) |
| Tir--Wor | 0.551 | 100 |  | Sad--Wor | 2.084 | 100 | Y (1.058) |
| Vis--Dei | 0.103 | 70 |  | Sle--Anh | 0.586 | 100 | Y (0.297) |
| Vis--Psi | 3.551 | 100 |  | Sle--Cnc | 1.194 | 100 | Y (0.6) |
| Voi--Dei | 0.102 | 80 |  | Sle--Dea | 0.265 | 100 | Y (0.144) |
| Voi--Psi | 2.216 | 100 |  | Sle--Dei | 0.485 | 100 | Y (0.255) |
| Voi--Vis | 2.191 | 100 |  | Sle--Sad | 2.649 | 100 | Y (1.305) |
| Wei--Dei | 0.3 | 100 |  | Sle--Tir | 0.969 | 100 | Y (0.493) |
| Wei--Psi | 0.222 | 80 |  | Sle--Wei | 0.949 | 100 | Y (0.479) |
| Wei--Vis | 0.117 | 90 |  | Tir--Dea | 0.231 | 100 | Y (0.128) |
| Wor--Con | 0.854 | 100 |  | Tir--Dei | 0.577 | 100 | Y (0.299) |
| Wor--Dea | 0.361 | 100 |  | Tir--Wei | 0.629 | 100 | Y (0.323) |
| Wor--Dei | 0.788 | 100 |  | Tir--Wor | 0.537 | 100 | Y (0.277) |
| Wor--Psi | 0.523 | 100 |  | Trau--Gre | -0.041 | 90.6 | N |
| Wor--Voi | 0.272 | 100 |  | Trau--IMD | 0.315 | 100 | Y (0.103) |
| Wor--Wei | 0.078 | 90 |  | Trau--Pol | 0.202 | 100 | Y (0.031) |
|  |  |  |  | Trau--PRd | 0.108 | 100 | Y (0.036) |
|  |  |  |  | Trau--PRs | 0.093 | 100 | Y (0.034) |
|  |  |  |  | Vis--Psi | 3.346 | 97.2 | Y (1.662) |
|  |  |  |  | Vis--Trau | 0.687 | 100 | Y (0.283) |
|  |  |  |  | Voi--Psi | 2.105 | 97.2 | Y (1.072) |
|  |  |  |  | Voi--Trau | 0.542 | 100 | Y (0.262) |
|  |  |  |  | Voi--Vis | 2.088 | 100 | Y (1.004) |
|  |  |  |  | Wei--Dei | 0.275 | 100 | Y (0.142) |
|  |  |  |  | Wei--IMD | 0.084 | 98.1 | Y (0.023) |
|  |  |  |  | Wei--Psi | 0.18 | 78.1 | Y (0.105) |
|  |  |  |  | Wei--Trau | 0.194 | 100 | Y (0.106) |
|  |  |  |  | Wei--Vis | 0.11 | 87.6 | Y (0.049) |
|  |  |  |  | Wor--Con | 0.824 | 100 | Y (0.37) |
|  |  |  |  | Wor--Dea | 0.328 | 100 | Y (0.161) |
|  |  |  |  | Wor--Dei | 0.761 | 100 | Y (0.378) |
|  |  |  |  | Wor--IMD | 0.03 | 71.6 | N |
|  |  |  |  | Wor--PRd | 0.121 | 100 | Y (0.034) |
|  |  |  |  | Wor--PRs | 0.055 | 92.6 | Y (0.009) |
|  |  |  |  | Wor--Psi | 0.462 | 97.2 | Y (0.229) |
|  |  |  |  | Wor--Trau | 0.459 | 100 | Y (0.22) |
|  |  |  |  | Wor--Voi | 0.184 | 92.7 | Y (0.081) |
|  |  |  |  | Wor--Wei | 0.078 | 94.3 | Y (0.034) |

**Table S4. Bridge nodes identified in Step-2 MGM network.**

| Node | Bridge expected influence | Bridge nodes |
| --- | --- | --- |
| Sad | 3.900 |  |
| Anh | 3.089 |  |
| Tir | 1.220 |  |
| Wor | 5.527 | X |
| Dea | 3.333 |  |
| Wei | 1.967 |  |
| Sle | 0.908 |  |
| Cnc | 1.566 |  |
| Dei | 5.109 | X |
| Con | 6.848 | X |
| Com | 2.447 |  |
| Voi | 4.185 |  |
| Vis | 5.082 |  |
| Psi | 8.555 | X |
| Trau | 8.734 | X |
| IMD | 0.905 |  |
| Pol | 0.092 |  |
| Gre | 0.009 |  |
| PRd | 0.922 |  |
| PRs | 0.364 |  |


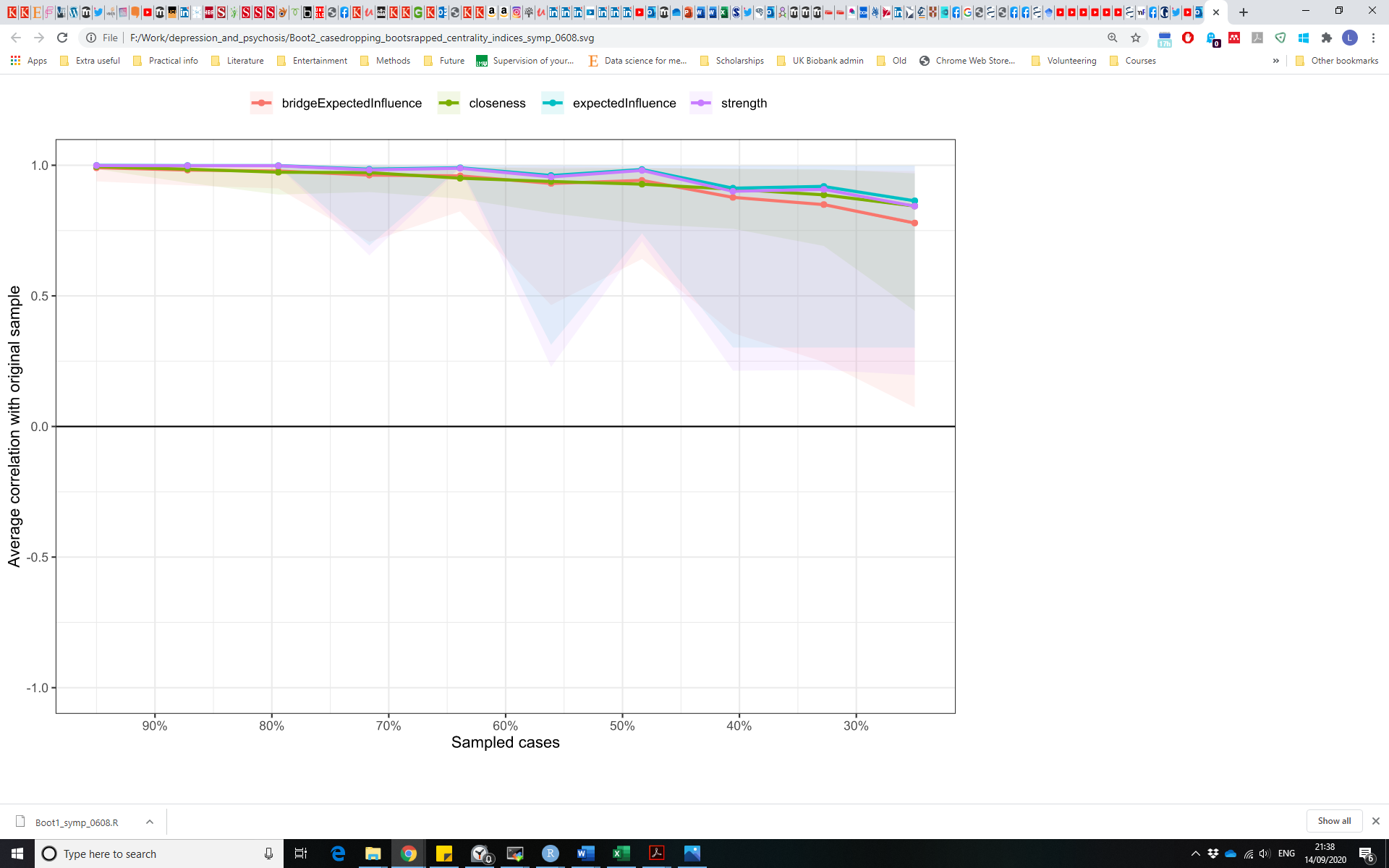


**Figure S1. Case-dropping bootstrapped centrality indices for Step-1 network.**

Correlation stability (CS) coefficients were equal to 0.75 for betweenness, 0.75 for closeness, 0.75 for strength, 0.75 for expected influence and 0.67 for bridge expected influence.


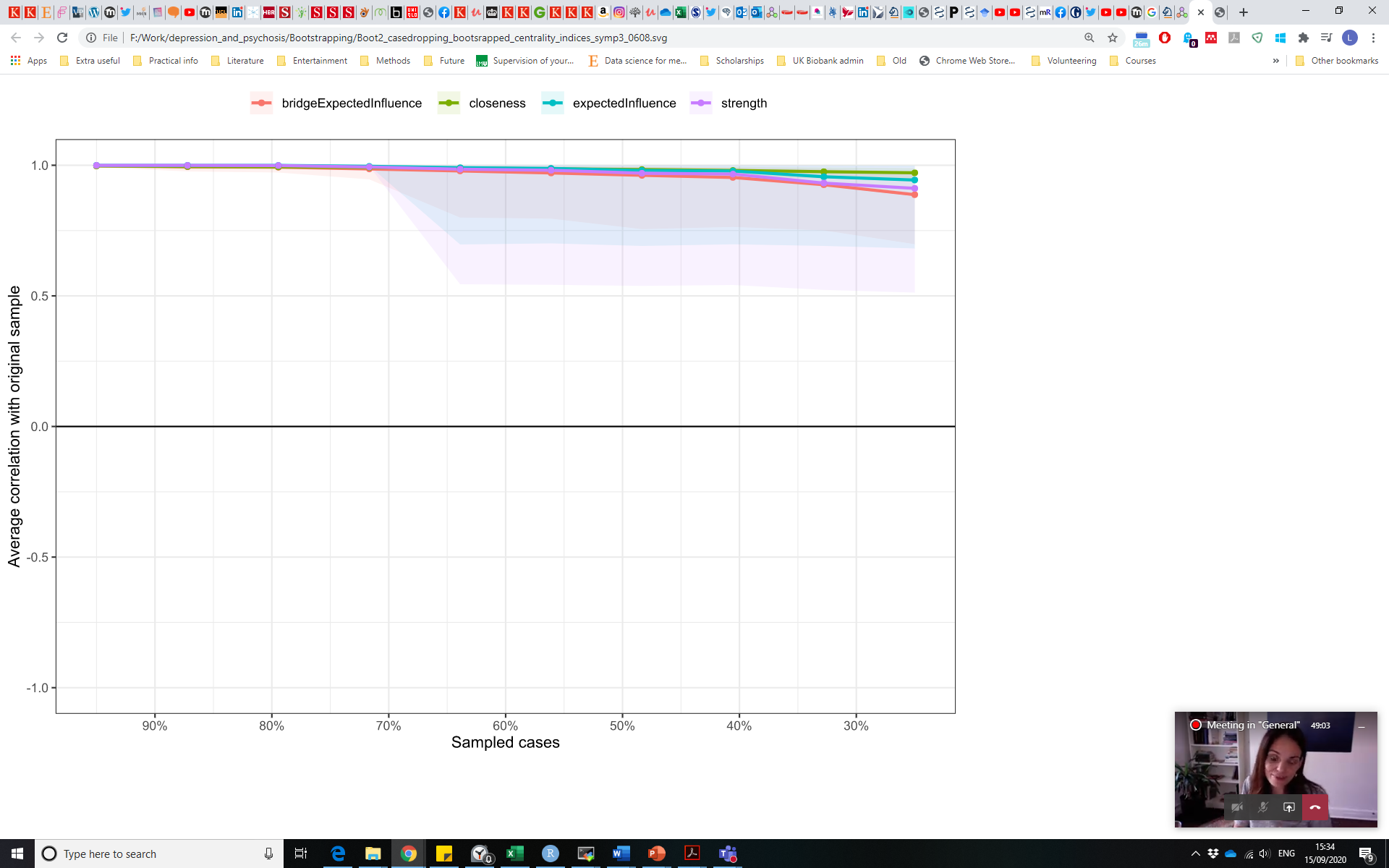


**Figure S2. Case-dropping bootstrapped centrality indices for Step-2 network.**

Correlation stability (CS) coefficients were equal to 0.59 for betweenness, 0.44 for closeness, 0.44 for strength, 0.59 for expected influence and 0.75 for bridge expected influence.


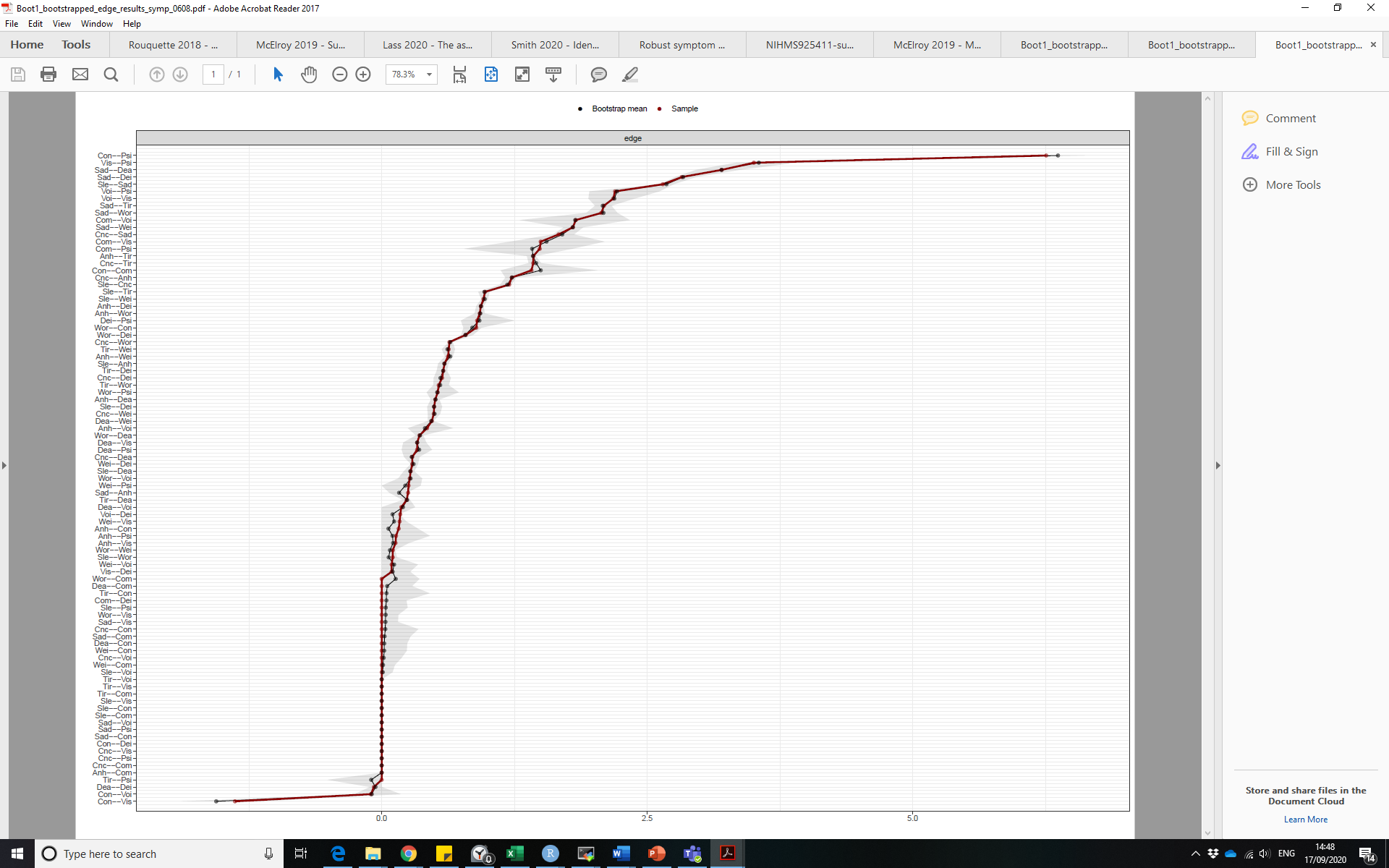


**Figure S3. Bootstrapped 95% confidence intervals for edge weights in Step-1 network.** The red dots represent the value of each edge weight, and the grey lines represent their bootstrapped 95% confidence interval.

#
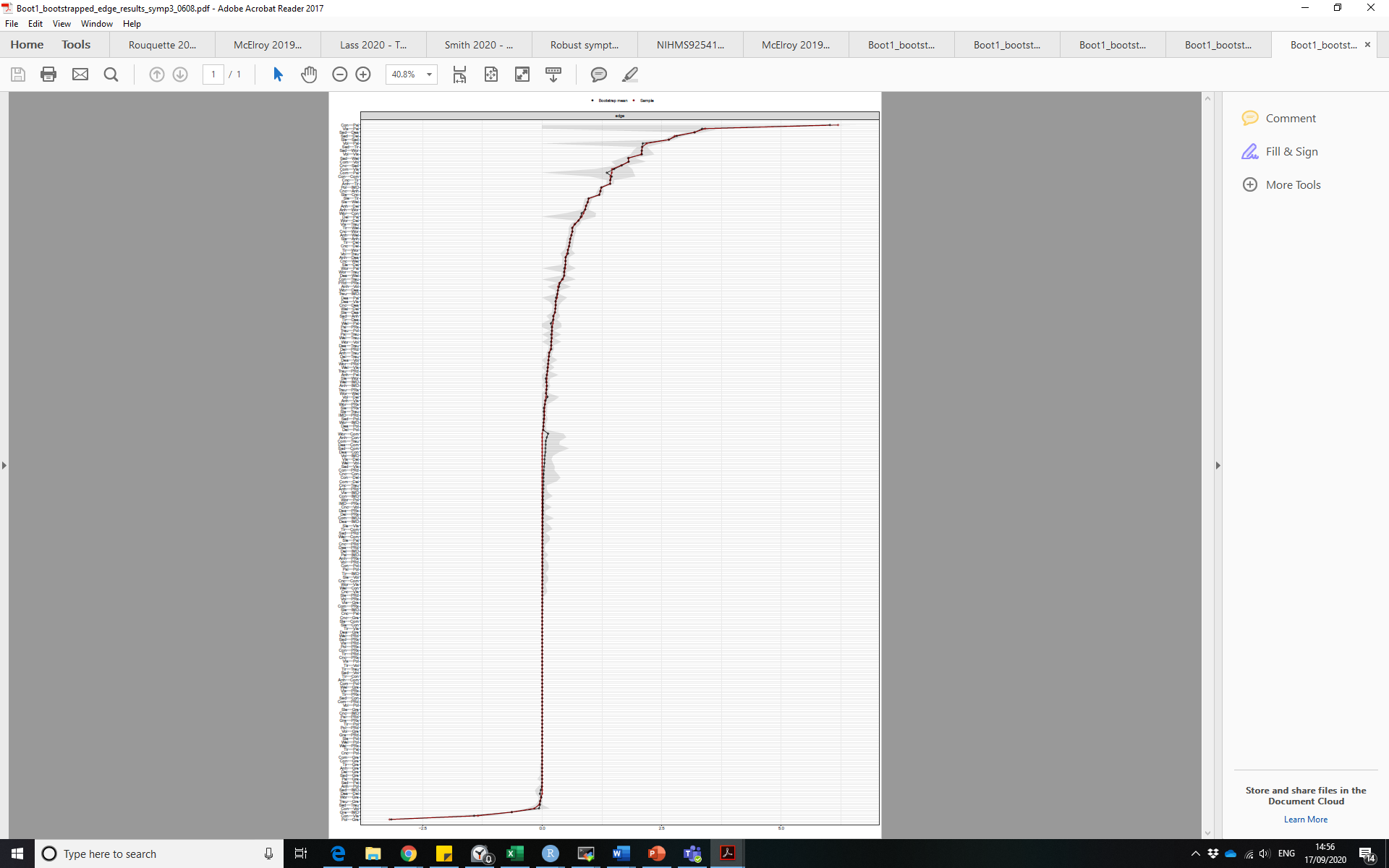


**Figure S4. Bootstrapped 95% confidence intervals for edge weights in Step-2 network.** The red dots represent the value of each edge weight, and the grey lines represent their bootstrapped 95% confidence interval.

**Down-sampled networks**


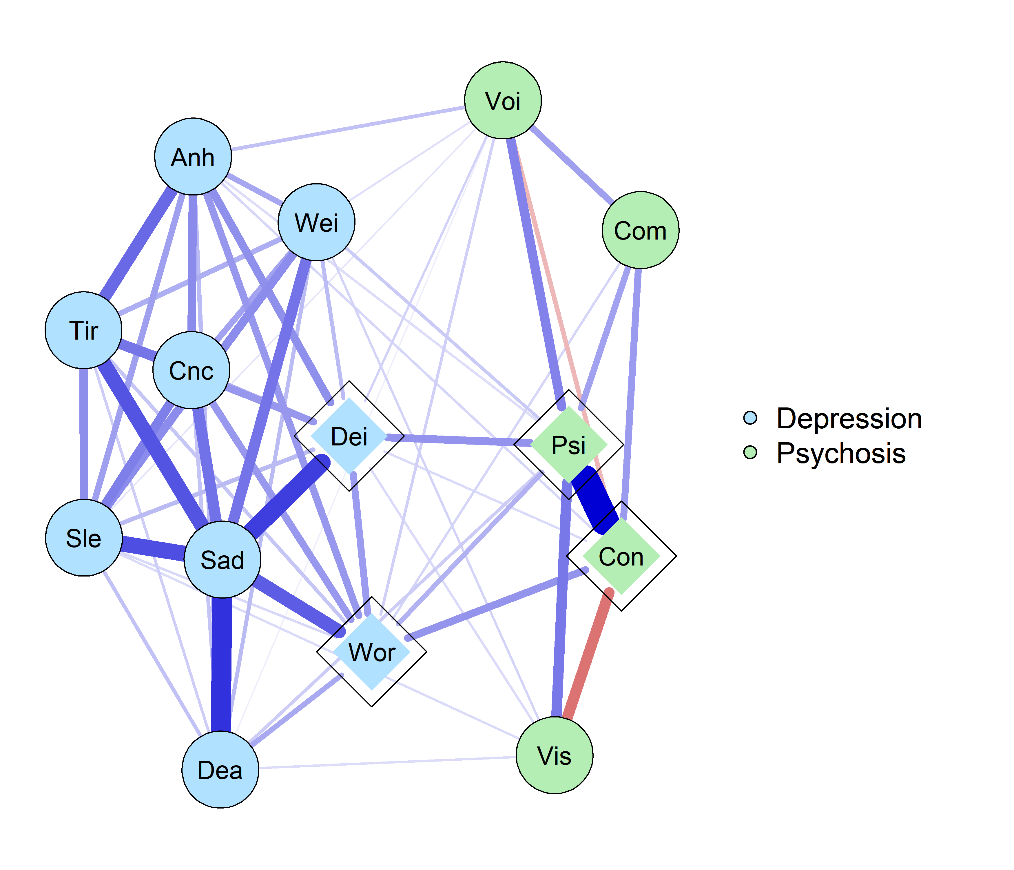


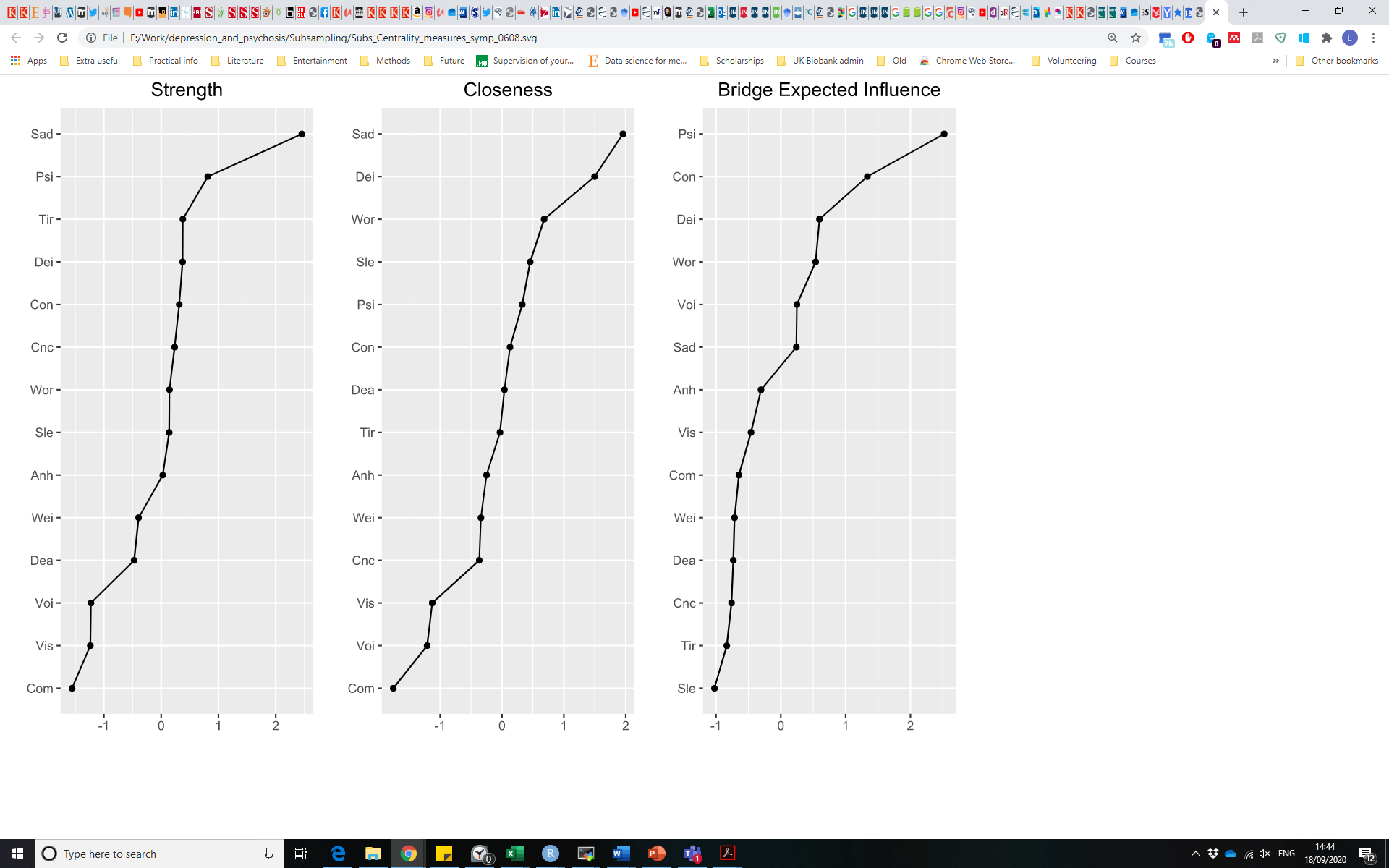


**Figure S5. Down-sampled Step-1 network with centrality indices and bridge expected influence index.**


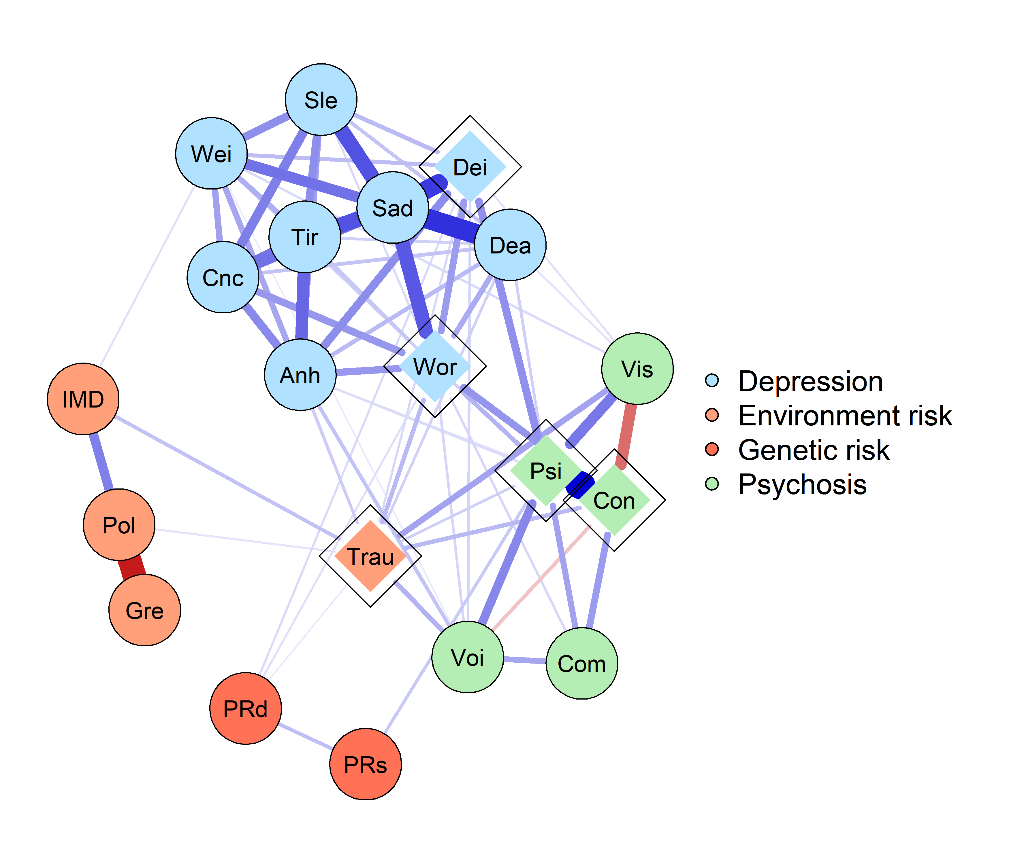


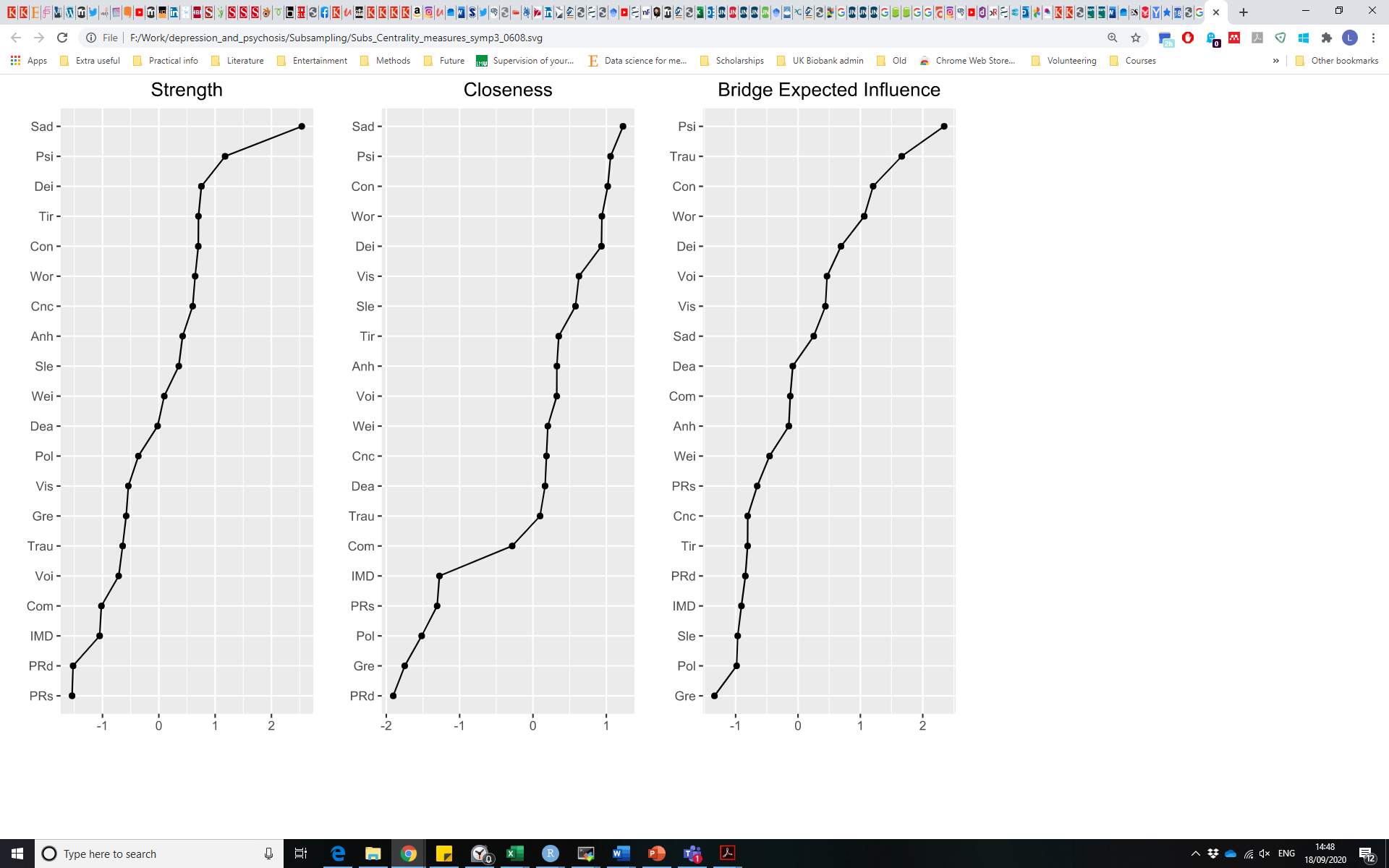


**Figure S6. Down-sampled Step-2 network with centrality indices and bridge expected influence index.**

**References**

1. Beelen R, Hoek G, Vienneau D, Eeftens M, Dimakopoulou K, Pedeli X, et al. Development of NO2 and NOx land use regression models for estimating air pollution exposure in 36 study areas in Europe – The ESCAPE project. Atmospheric Environment. 2013;72:10–23.

2. Office of the Deputy Prime Minister. Generalised land use database statistics for England. 2005.
